# The molecular mechanism and utilization of ZmMs7-mediated dominant nuclear sterility in *Oryza sativa* L

**DOI:** 10.3389/fpls.2025.1572721

**Published:** 2025-04-29

**Authors:** Yusheng Xu, Dingyang Yuan, Meijuan Duan

**Affiliations:** ^1^ School of Tea and Coffee, Pu’er University, Puer, China; ^2^ Yunnan International Joint Laboratory for Digital Conservation and Germplasm Innovation and Application of China-Laos Tea Resources, Pu’er University, Puer, China; ^3^ State Key Laboratory of Hybrid Rice, Hunan Hybrid Rice Research CenterAcademy of Agricultural Sciences, Hunan, Changsha, China; ^4^ Hunan Provincial Key Laboratory of Rice Stress Biology, College of Agronomy, Hunan Agricultural University, Changsha, China

**Keywords:** hybrid rice, foreign genes ZmMs7, dominant nuclear sterile lines, male-sterile line, transcriptome

## Abstract

**Introduction:**

Research on the molecular basis of dominant male sterility in rice and its application in sterile lines is significantly underdeveloped. This article aims to utilize dominant nuclear male sterile lines, which were created through the ectopic expression of *ZmMs7* in the genetic background of rice, for the purpose of heterosis utilization.

**Methods:**

At the same time, we conducted a study on the spatiotemporal expression characteristics of *ZmMs7*, performed transcriptome analysis, and implemented yeast two-hybrid experiments to elucidate its molecular regulatory mechanisms in mediating dominant nuclear male sterility in rice.

**Results:**

The results confirm the successful construction of a dominant nuclear male-sterile (NMS) vector system (p5126-ZmMs7-DsRed) using the exogenous male-sterile gene *ZmMs7*. This system comprises three modules: first, a dominant nuclear male-sterile (NMS) functional module driven by p5126, designed to achieve the dominant nuclear male-sterile trait; second, a fluorescence-based selection module driven by the endosperm-specific promoter LTP2, which facilitates the expression of the red fluorescent protein gene *DsRed*; and finally, a herbicide resistance screening module driven by the constitutive CaMV35S promoter, enabling the expression of the selectable marker *Bar* gene. The system has successfully developed a practical dominant male-sterile rice line characterized by complete pollen sterility, stable fertility, and straightforward visual seed selection, with no adverse effects on plant growth. In the hybrid offspring, approximately 50% of the seeds are genetically modified fluorescent seeds, while the remaining seeds are non-genetically modified and non-fluorescent. Transgenic plants Pro5126: GUS and ProZmMs7: GUS do not exhibit expression in roots, stems, leaves, or glumes. It is proposed that p5126 may enhance the expression of the *ZmMs7* gene, which could lead to the up-regulation of the rice pollen fertility gene *RIP1*, as well as the down-regulation of *OsMADS5* and the leafy glume sterile genes *OsMADS1* and *LHS1*.

**Discussion:**

Furthermore, it was demonstrated that the proteins encoded by these three fertility genes interact with the protein encoded by *ZmMs7*. This study provides new insights into the molecular regulatory network governing male reproductive development in rice and offers a theoretical foundation and technical support for the development of novel male-sterile germplasm resources.

## 1 Introduction

In the process of agricultural modernization, seeds are regarded as the foundation of agricultural development, and breeding high-quality rice varieties is crucial for ensuring food security in our country (Xing et al., 2020). “Sexual hybridization” is a breeding technique that involves crossing parents with distinct genetic backgrounds to produce superior recombined traits or create favorable variations. This technology has been extensively applied in the genetic improvement of both conventional rice and hybrid rice parents (Fu et al., 2020). Rice is a self-pollinating crop, necessitating artificial emasculation during the breeding process to prevent self-fertilization (Jiang et al., 2019). However, traditional manual emasculation and hybridization are labor-intensive and inefficient, posing challenges in meeting the demands of commercial rice breeding. The development of a new type of dominant male-sterile line would eliminate the need for tedious and complex manual emasculation processes, thereby facilitating direct hybridization with high-quality donor materials. Consequently, exploring and utilizing unique germplasm resources to reduce artificial emasculation in the sexual hybridization system is of great significance for enhancing the efficiency of rice breeding.

Nuclear gene-controlled male sterility in plants refers to a state of infertility that is entirely determined by nuclear genes, without any influence from cytoplasmic genetic variations. Current research widely recognizes two types: dominant nuclear male sterility and recessive nuclear male sterility, with the majority of cases falling under the latter category (Yu and Zhang, 2019). Male sterile lines serve as essential genetic materials for hybrid rice seed production. However, due to the limited application of male sterile lines induced by exogenous genes during rice breeding, their utilization remains scarce. The primary reason for this limitation is that the main factor leading to male sterility in rice involves recessive genes, while hybrid plants typically exhibit normal fertility. This scenario simplifies only the production process of hybrid seeds (F1 generation). In contrast, utilizing dominant nuclear male-sterile materials presents an excellent strategy for sexual hybrid breeding; these materials display male sterility characteristics in both homozygous and heterozygous genotypes. This approach not only eliminates the necessity for manual emasculation—thereby streamlining the sexual hybrid breeding process—but also facilitates rapid aggregation and efficient utilization of multi-gene germplasm resources. Consequently, when developing a new model for rice breeding, employing dominant nuclear male-sterile materials represents an effective means to overcome the challenges associated with artificial emasculation breeding technology (Yang et al., 2010).

Currently, there is a notable shortage of reported dominant nuclear sterile materials in rice, and both the application and fundamental research in this area are relatively underdeveloped. Researchers have identified five types of temperature-sensitive or incompletely sterile materials, including “Pingxiang dominant nuclear sterile” rice and the low-temperature sensitive variety known as “8987.” Both varieties demonstrate self-pollination at elevated temperatures. The success rates for “1783” and “1789” range from 0.3% to 3.5%, while “Zhe 9248M1” achieves a success rate of up to 24% (Deng and Zhou, 1994; Yan and Cai, 1997; Shu et al., 2000; Zhu, 2000). The utilization of dominant nuclear male-sterile mutants as tools for harnessing heterosis in rice encounters several challenges, including unstable fertility, difficulties in selectively separating hybrid seeds, and inefficient hybrid seed production. Dominant nuclear male sterile materials such as SMS, W450, OsDMS-1, and OsDMS-2 show promise as intermediate breeding tools for traits that are temperature-insensitive and completely sterile. However, the challenges associated with selecting seeds from male sterile lines present significant obstacles that diminish the breeding application value of temperature-insensitive dominant nuclear male sterile materials (Huang et al., 2008; Yang et al., 2017; Yi et al., 2017; Liang et al., 2018). Research has demonstrated that “SMS rice,” “Pingxiang dominant nuclear male sterility,” and 8987 are all governed by single genes, with their respective target genes located on chromosomes 6, 8, and 10. However, due to the inherent differences between dominant and recessive inheritance mechanisms, traditional methodologies for studying recessive traits have proven inadequate for cloning the single-gene control of dominant male sterility genes. The genetic control mechanisms of OsDMS-1 and OsDMS-2 are notably complex; specifically, the male sterility loci involve three gene loci in one case and two in another, while the fertility genes remain uncharacterized (Li et al., 1999; Yang et al., 2012). Based on comprehensive research findings to date, several challenges persist regarding the stability of male sterility in dominant nuclear sterile materials within rice. These challenges include incomplete pollen abortion as well as difficulties associated with seed propagation and selection. Such issues significantly hinder the practical application of dominant nuclear sterile rice in agricultural production. Furthermore, complications arising from gene cloning difficulties and relatively intricate genetic mechanisms have contributed to a slow advancement in understanding the molecular regulatory frameworks governing dominant nuclear sterile rice.

The ectopic expression of male fertility genes and fluorescent protein genes can be employed to develop practical dominant nuclear sterile lines in rice, thereby facilitating the observation and selection of their progeny. Similar to the dominant nuclear male sterility resulting from endogenous gene mutations, ectopic expression may disrupt male organ development, leading to characteristics associated with dominant nuclear male sterility. Research findings indicate that the overexpression of *AtMs1* and *HvMs1* induces dominant nuclear male sterility in Arabidopsis thaliana and barley (Gómez and Wilson, 2014). The maize gene *ZmMs7* exhibits a function analogous to that of *AtMs1* and *HvMs1*, playing a critical role in pollen exine formation and tapetum development by encoding PHD zinc finger transcription factors (Zhang et al., 2018).

The normal development of the tapetum layer significantly influences male reproductive capability. Research conducted by our group on maize indicates that the anther-specific promoter p5126 exhibits specific expression in the tapetum layer during stages 7 to S8a, which occurs earlier than the activation period of the *ZmMs7* gene’s own promoter (stages 8b to 10). Transcriptome sequencing and protein interaction experiments have demonstrated that *ZmMs7* directly regulates maize silk development by activating ZmMT2C. Fluorescent quantitative PCR has confirmed that the expression of ZmMs7 in dominant nuclear male-sterile maize is one to two stages ahead of that in wild-type plants, resulting in the manifestation of dominant nuclear male sterility (An et al., 2020). In rice, either delayed or premature programmed cell death within the tapetum layer can also lead to male sterility. For instance, mutations in genes such as *UDT1*, *PTC1/OsMS1*, and *PTC2* can delay tapetum degradation, leading to male nuclear sterility; conversely, a single base variation in pms3 causes premature programmed cell death of the tapetum layer and results in photoperiod-sensitive male nuclear sterility (Jung et al., 2005; Li et al., 2011; Ding et al., 2012; Uzair et al., 2019). Given the differences between male sterility caused by dominant nuclear genes and that resulting from recessive genetic factors, as well as its distinction from the dominant nuclear male sterility regulated by *ZmMs7* in maize. It can be inferred that the exogenous gene *ZmMs7* mediates a novel mechanism affecting programmed cell death in rice anther tapetum cells, leading to dominant male sterility in rice. However, the molecular mechanism by which *ZmMs7* mediates the development of rice anther tapetum cells is not yet clear and requires further investigation.

**Figure 1:**
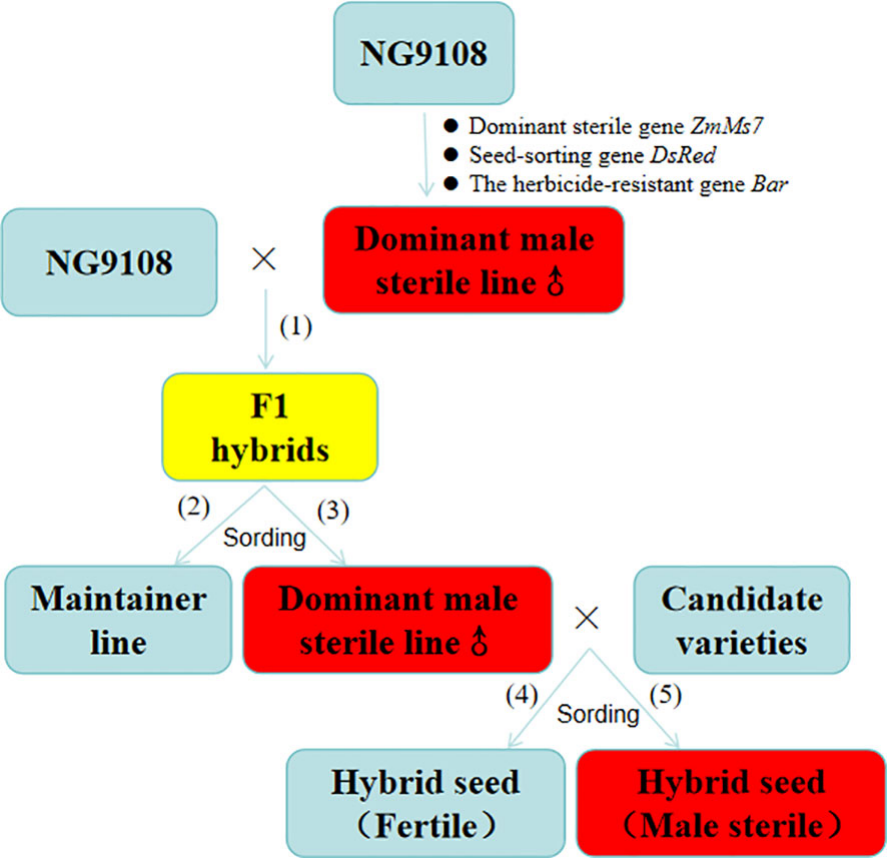
The reproduction of sterile lines and maintainer lines, as well as the production of hybrid F1 seeds (1): Production of F1 hybrids, (2): Production of maintainer line, (3): Production of Dominant male sterile line, (4): Seeds without fluorescence, (5): Seeds with fluorescence.

In this study, the p5126 pollen-specific promoter was utilized to drive the ectopic expression of the maize gene *ZmMs7*, resulting in the successful development of a dominant nuclear male-sterile line in maize. Using a similar approach, we employed the “Nangeng 9108” rice background to construct a dominant male-sterile system and generated a novel rice material, p5126-ZmMs7-DsRed-YS, with dominant nuclear male sterility (**Figure 1**). Concurrently, research will be conducted on the spatiotemporal expression characteristics, transcriptome analysis, and yeast hybridization of the *ZmMs7* dominant nuclear male-sterile line to analyze the molecular regulatory mechanism of rice male sterility mediated by *ZmMs7*. This study provides new insights into the molecular regulatory network governing male development in rice, and offer theoretical basis and technical support for the creation and utilization of new sterile germplasm resources. At the same time, the data obtained can serve as a reference for the production and application of other dominant nuclear sterile materials in plants.

## 2 Materials and methods

### 2.1 Creation of indica dominant nuclear male sterile lines in rice

#### 2.1.1 Acquisition of materials

The maize anther-specific promoter p5126 was used to construct a male-sterile system, p5126-ZmMs7-DsRed, by introducing exogenous genes (*ZmMs7* from maize, *DsRed* fluorescent protein gene, and the herbicide-resistant *Bar* gene). This construct was then transformed into the recipient material of rice (the 2021 variety “Nangeng 9108” (NG9108)), resulting in the generation of transgenic rice materials. In the T0 generation, 10 sterile mutant samples were collected. These sterile samples, after backcross with the NG9108 bagging kit, were named as the p5126-ZmMs7-DsRed-YS sterile line due to their non-fruiting rate.

#### 2.1.2 Experimental materials and planting

The experiment conducted at Chunhua Base in Changsha involved a single plant reproduction test on 14 rice samples at the T0 stage. The rice plants are cultivated using a double-row planting method, with seven plants per row. The inter-row spacing is set at 25 centimeters, while the intra-plant spacing is maintained at 20 centimeters. The same method was also employed for the planting of the T1 generation. A total of 40 samples were used for the single plant breeding experiment, with half from the Ya Zhou base in Hainan and the other half from the Chunhua base in Changsha. The T1 generation materials are distributed using a four-row planting pattern, with each row containing five plants. The sowing density is consistent with that of the T0 generation. All field management work followed conventional field fertilization and irrigation methods (Ma et al., 2021).

#### 2.1.3 Investigation of T0 generation reproductive and agronomic traits

In the field, precise measurements of the above-ground height of rice plants were conducted using a ruler. Simultaneously, visual observations were employed to study the characteristics of rice grains in terms of shape and size, as well as the features of anthers. Preliminary observations were made under fluorescent light to determine if the grains exhibit fluorescence characteristics. Subsequently, unopened panicles were brought back to the laboratory for microscopic observation of anther morphology and detection of pollen activity.

### 2.2 Mechanism of dominant nuclear male sterility driven by P5126-ZmMs7-DsRed in rice

#### 2.2.1 GUS staining

Construct pC1301-p5126::GUS and pC1301-pZmMs7::GUS vectors to drive the expression of *GUS* gene, and transform them into rice with the same background. After obtaining transgenic *GUS* lines, perform GUS staining on roots and young panicles at different stages to determine the expression pattern of the gene in different periods and tissues (Wang et al., 2013). Utilizing laser confocal microscopy to observe the luminescent localization of green fluorescent protein within cells, in order to determine the subcellular localization of *ZmMs7* in dominant nuclear male-sterile rice. Designing fluorescent probes targeting *ZmMs7* for *in situ* hybridization with the target RNA, and analyzing the spatial expression information of *ZmMs7* mRNA in tissue cells after signal amplification.

#### 2.2.2 qRT-PCR detection

According to the instructions for use of the reagent, the TRIzol method was used to extract total RNA from rice leaves. In this experiment, the extracted RNA will be converted into cDNA using a reverse transcription reagent kit from Thermo Fisher Scientific. Detailed operating procedures can be found in the instruction manual of the kit. qRT-PCR experimental reaction conditions: Initially, incubate at 50 °C for two minutes, followed by a 10-minute preheating at 94 °C. Subsequently, perform a 5-second heating at 94 °C and a 30-second heating at 60 °C, followed by 40 cycles. Throughout this process, fluorescence signals are collected at a single point while maintaining a constant temperature of 60 °C. As an internal reference control, UBQ (*LOC_Os03g13170*) was selected, and the experiment was repeated three times. Ultimately, gene expression levels were determined using the 2-ΔΔCt method (Yang et al., 2019).

#### 2.2.3 Methods for transcriptome analysis of rice panicles at different developmental stages

**Differential gene screening:** The gene counts of each sample were standardized and the differential fold changes were calculated using the DESeq2 software. Subsequently, the negative binomial distribution (NB) method was employed to detect the significance of differences. Finally, based on the results of differential fold changes and significance testing, different protein-coding genes were selected. The default criteria for selection are a q-value less than or equal to 0.05 and a fold change exceeding 1.5 (An et al., 2020).

**Clustering analysis of differential gene expression levels:** A distance matrix was constructed using the distance values between samples, and similar categories were combined into new categories. Subsequently, the distances between the new and existing categories were compared, and the process of combination and calculation continued until only one category remained (Chen et al., 2022). The protein-coding genes with significant differences were selected as references to evaluate the degree of relationship between samples.

**Differential gene GO enrichment analysis:** Firstly, all sample sequences that are different from the normal state are statistically counted. Then, these sequences are input into the software system to obtain the number of relevant differential genes. The document is imported into the computer program and the significance of differential gene enrichment is calculated using the hypergeometric distribution algorithm. Finally, Fisher’s exact test can accurately calculate the significance P value for each term in BP, CC, and MF. Based on the results of GO analysis combined with biological significance, suitable genes for subsequent research are selected (Wang et al., 2022). The formula for calculating p-value in hypergeometric distribution test Equation 1 and enrichment score calculation formula Equation 2 are as follows:


(1) $$p=1-\sum_{i=0}^{m-1}\frac{\left(\frac{M}{i})(\frac{N-M}{n-i}\right)}{\left(\frac{N}{n}\right)}$$

(2) Enrichment score=mnMN

Note: Let N represent the total number of genes with GO annotation, n represent the number of differentially expressed genes with GO annotation among N, M represent the total number of genes labeled with a specific GO term among all genes, and m refer to the number of genes that are GO annotated and differentially expressed with the specific GO term.

Utilizing the KEGG database for pathway analysis of differentially expressed protein-coding genes, and employing the hypergeometric distribution test to calculate the significance of enrichment of differential genes in each pathway entry.

**Fluorescent quantitative PCR detection:** Four candidate differentially expressed genes that may regulate and influence the normal development of rice anthers were selected for quantitative detection. The fluorescent quantitative PCR experiment was conducted using the PerfectStartTM Green qPCR SuperMix kit on the LightCycler^®^ 480 II fluorescent quantitative PCR instrument. Finally, the expression level was calculated using the 2^-ΔΔCt^ method, as shown in Equation 3 (Hai et al., 2022):


(3) $$\begin{array}{c} 2^{-\Delta \Delta \text{Ct}}=2^{-(\Delta {\text{Ct Samples from the experimental group}}^{-}\Delta \text{Ct Control group samples})}\\ =2^{-\Delta \text{Ct Control group samples}}/2^{-\Delta \text{Ct Samples from the experimental group}} \end{array}$$

Note: The ΔCt is calculated by subtracting the Ct value of the reference gene from the Ct value of the target gene, while ΔΔCt represents the difference between the ΔCt values of the experimental group samples and those of the control group samples.

#### 2.2.4 Yeast two-hybrid experiment

**pGBKT7-ZmMs7 domain nuclear body system dual-hybrid self-activation experiment:** Using plasmids pGBKT7-Lam and pGADT7-T as negative controls, and plasmids GBKT7-53 and pGADT7-T as positive controls (Kim et al., 2018). The Y2HGold yeast strain is cultured, then evenly spread onto a 100 mm wide plate according to the data in **Supplementary Table 1**. In this experiment, the Positive control, Negative control, three types of BD self-activation detection, and the Blank BD Carrier group were established, and compared with the expected results according to the data in **Supplementary Table 1**.

**Co-transformation - Double Hybrid Library Screening** (Day et al., 2006): The bacterial cells suspended in a 0.9% NaCl solution were added to the original solution, resulting in a total volume of 6 mL. Subsequently, 100 μL of both the tenfold and hundredfold dilutions were evenly spread on SD/-Trp/-Leu plates with a diameter of 100 mm for the purpose of calculating the Transformation Efficiency. The specific Equation 4 are as follows:


(4) $$\text{Transformation Efficiency}=\frac{\text{cfu x suspensiion volume}}{\text{volume of plated x amount of DNA}}$$

Spread the unused bacterial solution evenly on SD/-Trp/-Leu/Xa-Gal/AbA plates, 150 μL per plate, with approximately 50 plates in total. Incubate the plates at 30 °C for about 35 days until 1-2 mm monoclonal colonies appear, completing the primary screening. Transfer the blue positive clones from the primary screening plates to secondary selection medium SD/-Trp/-Leu/-His/-Ade/X-a-Gal/AbA for more rigorous screening (secondary screening). Incubate at 30 °C for 3-5 days and select those with blue positive growth for one-on-one validation.

## 3 Results

### 3.1 Successful creation of a new type of male-sterile line with dominant genic male sterility in rice

#### 3.1.1 Construction of Pro5126-ZmMs7-DsRed vector system

**Figure 2:**
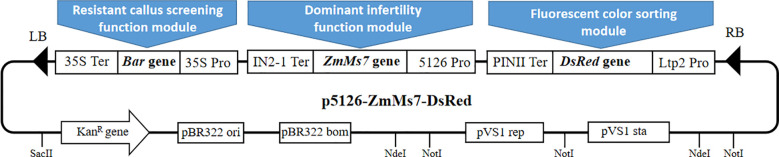
Pro5126-ZmMs7-DsRed vector system.

The visualized sorting dominant male sterile carrier system Pro5126-ZmMs7-DsRed comprises three functional modules (**Figure 2**): the *ZmMs7* expression cassette, driven by the p5126 promoter, which imparts dominant nuclear male sterility to plants (the dominant male sterile functional module); the red fluorescent protein gene *DsRed*, regulated by the endosperm-specific promoter LTP 2, serving as a screening marker for seeds derived from dominant nuclear male sterile materials (the fluorescence selection module); and the herbicide-resistant *Bar* gene, governed by the CaMV35S constitutive promoter, utilized as a screening marker for transgenic callus exhibiting dominant nuclear male sterility (resistance callus screening functional module). This carrier system is anticipated to facilitate the development of sterile lines across various plant species, offering broad and reliable prospects for application.

#### 3.1.2 Phenotypic Characteristics of Novel Dominant Male Sterile Materialss

**Figure 3:**
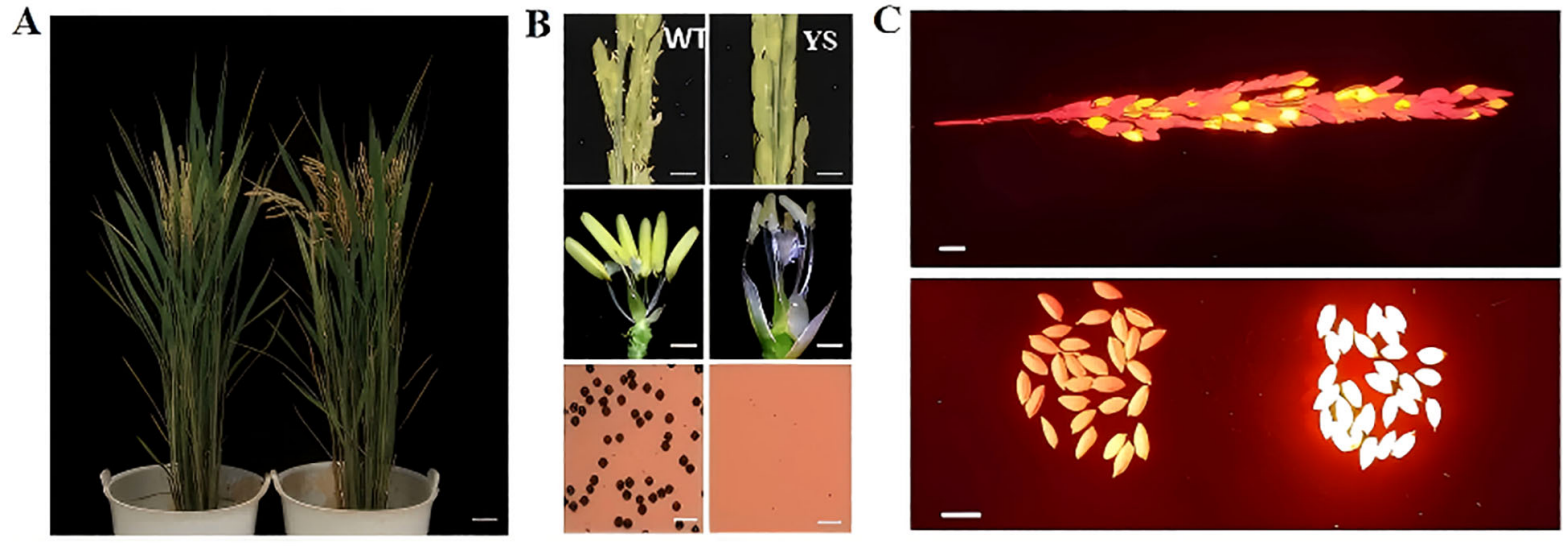
The male sterile phenotype characteristics of p5126-ZmMs7-DsRed-YS **(A)** Plant morphology of wild type and dominant nuclear sterile line p5126-ZmMs7-DsRed-YS at heading stage; **(B)** Plant morphology of wild type and dominant nuclear sterile line p5126-ZmMs7-DsRed-YS Ear, anther (Scale bars, 1 mm) and I2-KI stained pollen grains (Scale bars, 100 um); **(C)** After crossing the dominant nuclear sterile line with the wild type, the fluorescent seeds of the offspring (dominant nuclear sterility) Seeds) account for about 50% (Scale bars, 1 cm).

The dominant nuclear male sterile line p5126-ZmMs7-DsRed-YS is characterized by smaller anthers that produce no viable pollen, while maintaining normal female fertility and exhibiting no adverse effects on other traits (**Figures 3A, B**). Notably, the Pro5126-ZmMs7-DsRed element responsible for this dominant male sterility can only be inherited through the female gamete. Therefore, among the offspring produced by the hybridization of explicit nuclear sterile rice and wild-type rice, approximately 50% of the seeds exhibit fluorescent characteristics, while the remaining 50% do not display any fluorescence (see **Figure 3C**). The seeds with fluorescence are dominant genic male-sterile seeds. Such seeds show potential application value in hybrid breeding. And hybrid experiments were conducted using various rice varieties and sterile line. Among the obtained hybrid rice, the ratio of fluorescent seeds to non-fluorescent seeds was 1:1.

#### 3.1.3 Infertile Vector System is Stably Inherited to the Next Generation

**Figure 4:**
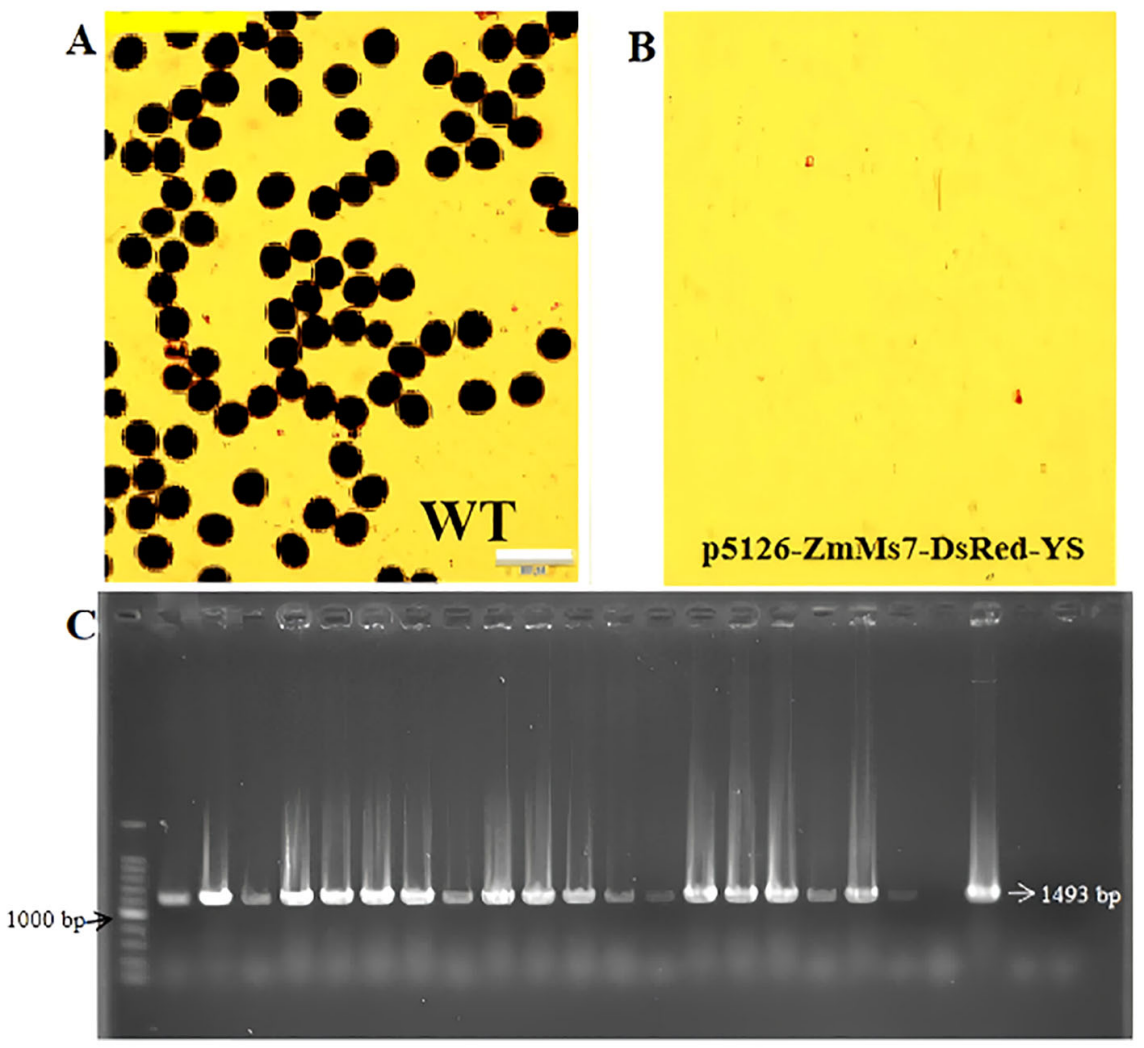
Male sterility identification of F1 generation transgenic materials **(A, B)** I2-KI stained pollen grains (Scale bars, 100 um) of wild-type and dominant nuclear sterile lines p5126-ZmMs7-DsRed-YS, **(C)** Primers of p5126 promoter (1493 bp) Amplified band diagram.

The F1 generation seeds obtained by crossing the T0 generation sterile line and NG9108 were screened, and the seeds with fluorescent characteristics were selected for planting. After sequencing and comparison, the results showed that the 1992nd base was A. After converting to G, it belonged to a synonymous mutation, and the corresponding amino acid was Serine. This indicates that the heterologous gene ZmMs7 has been successfully introduced into japonica rice NG9108. Collect the leaves of F1 generation plants for PCR identification and microscopic examination of anther and pollen fertility during the flowering period. The results showed that the anthers and pollen of F1 generation positive plants were inactive, while the wild-type plants had normally active pollen (**Figures 4A, B**). At the same time, the expected bands could be amplified by PCR detection (**Figure 4C**), indicating that the dominant male sterile vector system had been successfully inherited to the F1 generation. The subsequent dominant nuclear male sterile line was test crossed with 20 rice varieties. The 20 obtained combinations showed that the ratio of fluorescent seeds to non-fluorescent seeds was approximately 1:1.

### 3.2 Mechanism of dominant male sterility mediated by exogenous gene ZmMs7 in rice

#### 3.2.1 Pro5126 induces early expression of the ZmMs7 gene in anthers

**Figure 5:**
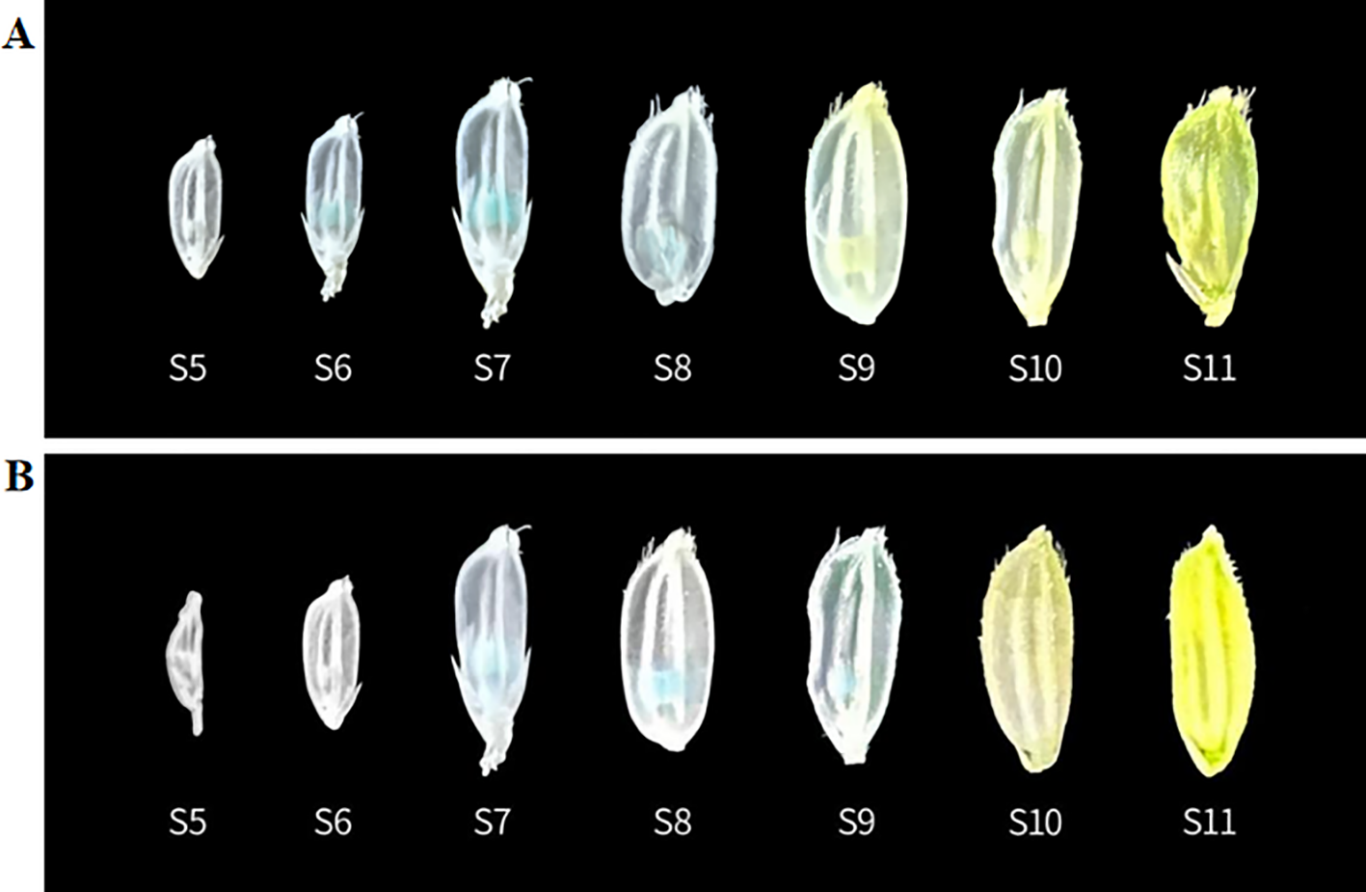
*GUS* staining of rice seeds at different periods **(A)** Pro5126: GUS transgenic plants; **(B)** ProZmMs7: GUS transgenic plants. Seed stages: S6 is the tapetum formation stage, S7 is the prophase of meiosis, S8 is the late stage of meiosis, and S9 is the microspore formation stage.

**Figure 6:**
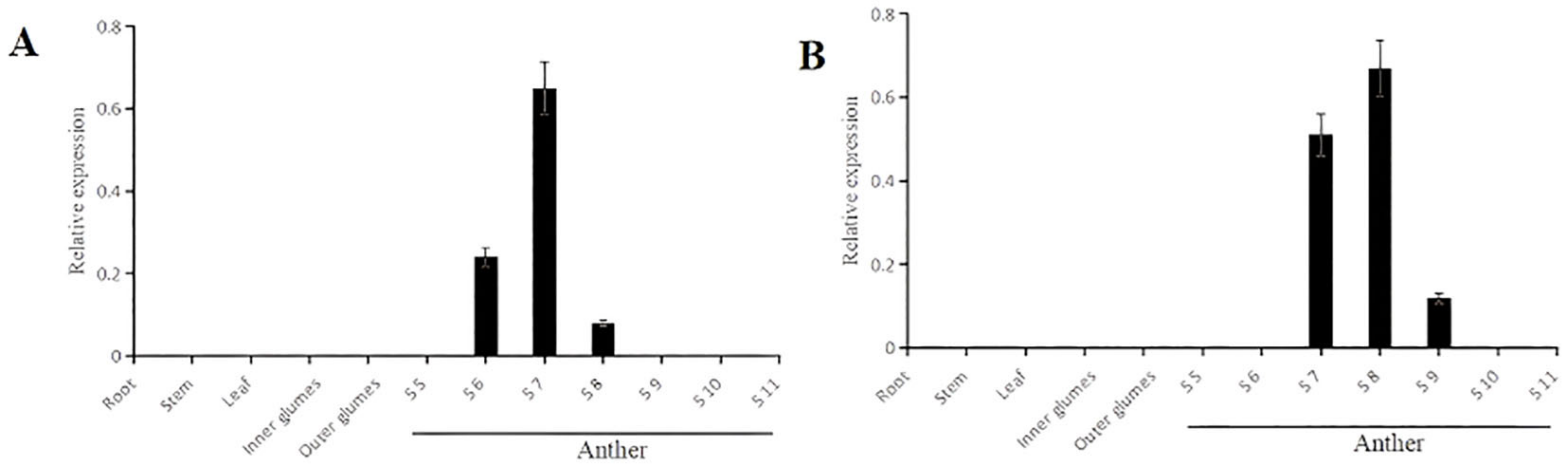
Pro5126: GUS and ProZmMs7: GUS transgenic plants qRT-PCR analysis of different tissues at various stages **(A)** Pro5126: GUS transgenic plants; **(B)** ProZmMs7: GUS transgenic plants. S6 is the tapetum formation stage, S7 is the prophase of meiosis, S8 is the late stage of meiosis, and S9 is the microspore formation stage.

The GUS staining was conducted on germinating seeds and 4-day-old seedlings of the transgenic plants Pro5126:GUS and ProZmMs7:GUS. No GUS signal was observed in either the germinating seeds or the roots (**Supplementary Figure 1**). In flowering-stage transgenic plants, GUS staining of leaves and seeds revealed that the signal was predominantly localized within the anthers. For Pro5126:GUS, the *GUS* signal during early anther development (tapetum formation period, S6) was weak, peaked at the pre-meiotic stage (S7), and subsequently diminished during the post-meiotic stage (S8) (**Figure 5A**). The transgenic plant ProZmMs7:GUS exhibited no *GUS* signal during early anther development, a strong signal at the pre-meiotic stage (S7), the strongest signal at the post-meiotic stage (S8), and a diminished signal during microspore formation (S9) (**Figure 5B**). It is hypothesized that Pro5126 promotes earlier expression of the *GUS* gene by one developmental stage. qRT-PCR analysis confirmed that both Pro5126:GUS and ProZmMs7:GUS showed no expression in roots, stems, leaves, inner glumes, or outer glumes. In anthers, Pro5126:GUS was expressed from stages 6 (tapetum formation) to 8 (late meiosis), peaking at a value of 0.65 in stage 7 (**Figure 6A**). Meanwhile, ProZmMs7:GUS reached its highest expression level of 0.67 in stage 8 (**Figure 6B**), further corroborating their presence in anthers. Compared to ProZmMs7:GUS, Pro5126 induces earlier *GUS* gene expression, suggesting it may advance *ZmMs7* expression.

#### 3.2.2 Abnormal expression of fertility-related genes in the tapetum leads to dominant male sterility in rice

**Figure 7:**
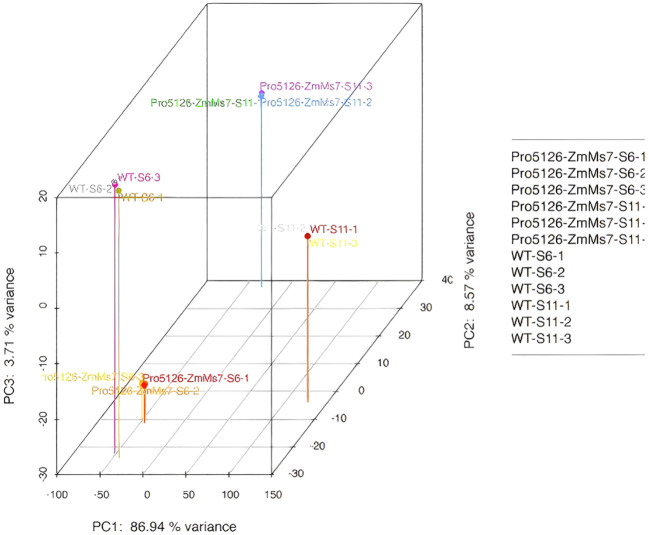
3D diagram of PCA.

**The samples are qualified and the sequencing data meets the quality requirements for analysis:** The 3D distances of each parallel sample are close, and there is a certain distance between different samples at each time point. The content of the highest principal component PC1 is 86.94% (**Figure 7**), further demonstrating the repeatability of biological experiments and the reliability of differential gene expression.

**Figure 8:**
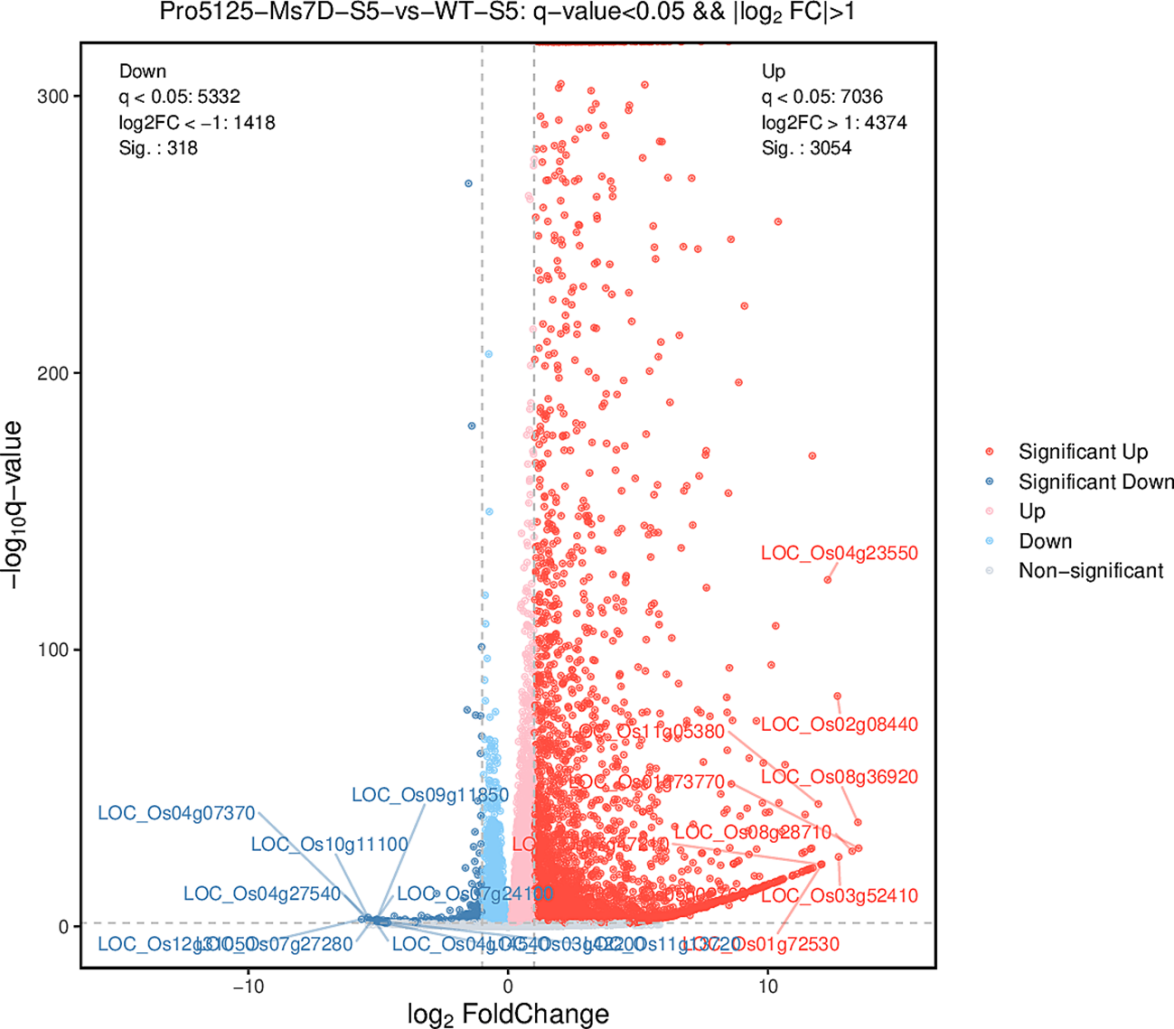
Volcano plot of differential expression between Pro5126-ZmMs7-S6 and WT-S6.

There are significant differences in the genetic data of transgenic and background materials over time. According to **Table 1**, at stage 6, the number of up-regulated differentially expressed genes in the transgenic material (Pro5126-ZmMs7-S6-VS-WT-S6) is markedly higher than that observed in wild-type material (4260 vs. 1092) (**Figure 8**). Notably, the total number of differentially expressed genes is at its lowest point (5352) during this stage. Furthermore, the numbers of both up-regulated and down-regulated genes exhibit similarities between the 11th generation transgenic material and wild-type material (Pro5126-ZmMs7-S11-VS-WT-S11), as well as between the 6th generation transgenic material and its preceding generation (Pro5126-ZmMs7-S11-VS-Pro5126-ZmMs7-S6).

**Table 1:** Comparison of differential genes.

Case	Control	Up_diff	Down_diff	Total_diff(q-value<0.05&|log2FC|>0.58)
Pro5126-ZmMs7-S11	WT-S11	6692	7525	14217
Pro5126-ZmMs7-S6	WT-S6	4260	1092	5352
Pro5126-ZmMs7-S11	Pro5126-ZmMs7-S6	6918	6961	13879

**Figure 9:**
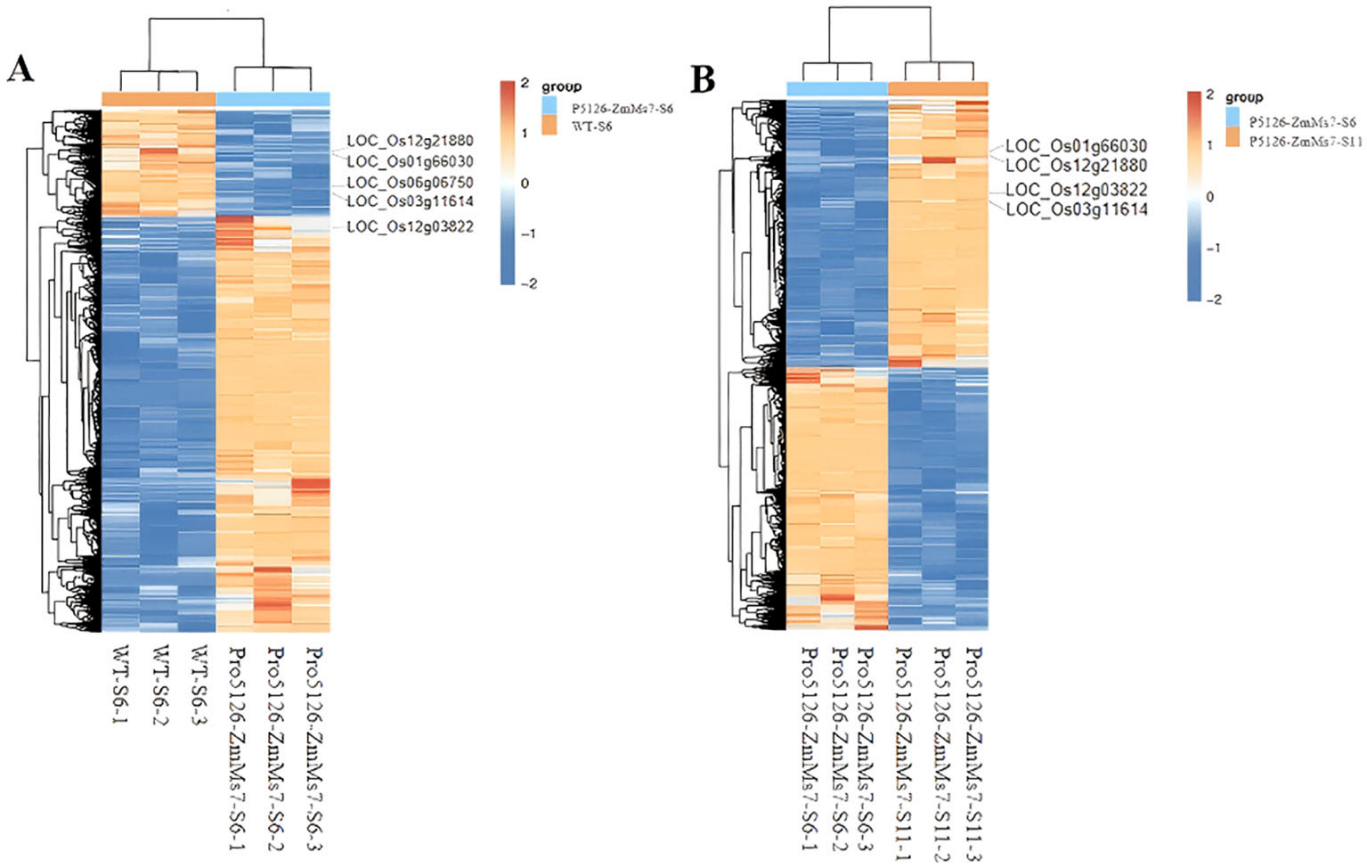
Differential gene grouping and clustering **(A)** Pro5126-ZmMs7-S6-VS-WT-S6 group, **(B)** Pro5126-ZmMs7-S11-VS-WT-S11 group. Red in the figure represents relatively highly expressed protein-coding genes, and blue represents relatively low-expressed protein-coding genes.

**P5126-ZmMs7 leads to abnormal expression of rice fertility genes during the meiotic stage in the tapetum layer:** According to **Table 1**, it is evident that Pro5126-ZmMs7-S6-VS-WT-S6 has more up-regulated genes than down-regulated genes. However, as shown in **Figure 8**, the genes related to fertility are mainly down-regulated. The main includes five fertility-related genes: *LOC_Os12g03822* (up-regulated), *LOC_Os12g21880* (down-regulated), LOC_Os06g06750 (down-regulated), *LOC_Os01g66030* (down-regulated), and *LOC_Os03g11614* (down-regulated) (**Figure 9A**). Compared to the S6 stage, the fertility genes *LOC_Os12g03822* (up-regulated), *LOC_Os12g21880* (up-regulated), *LOC_Os01g66030* (up-regulated), and *LOC_Os03g11614* (up-regulated) in Pro5126-ZmMs7-S11 show an increase in their expression levels during the later stages of fertility development (**Figure 9B**). It is speculated that p5126-ZmMs7-DsRed leads to overexpression or downregulation of fertility genes in the tapetum stage, resulting in male sterility in rice.

**Figure 10:**
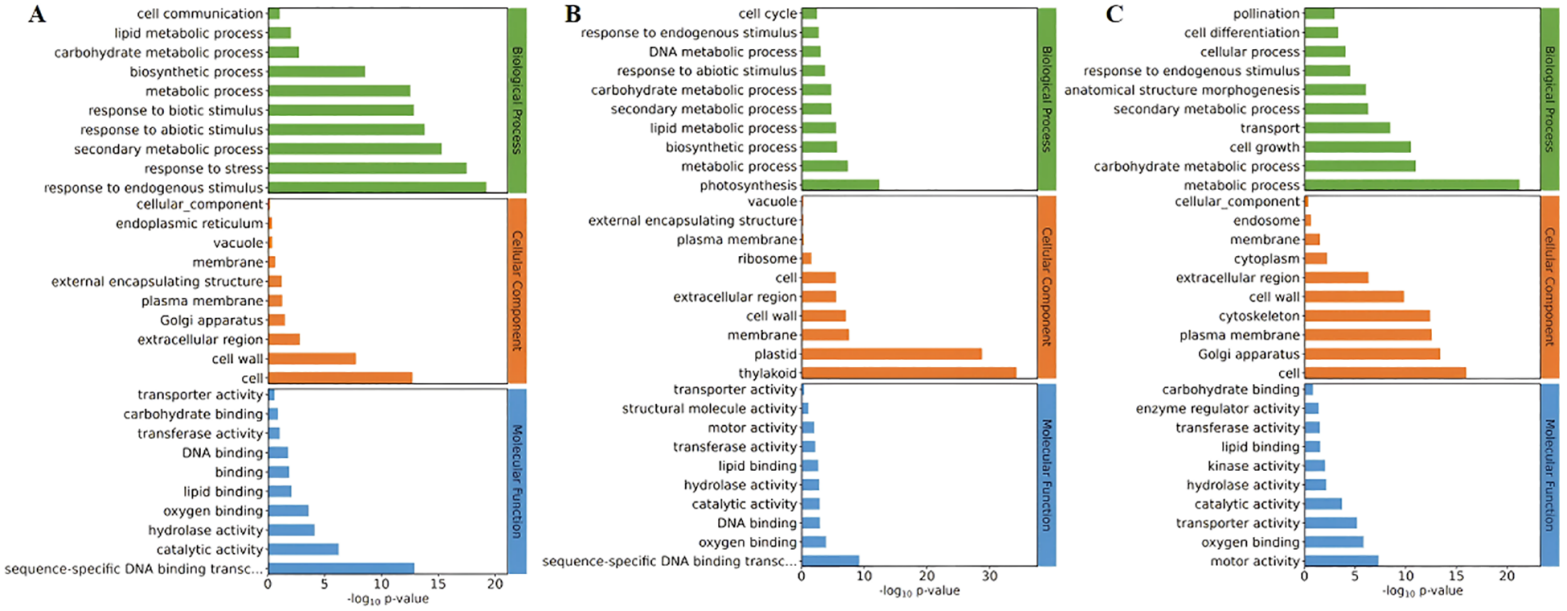
Comparison of GO enrichment among different rice materials **(A)** Pro5126-ZmMs7-S6-vs-WT-S6 (top 30), **(B)** Pro5126-ZmMs7-S11-vs-Pro5126-ZmMs7-S6 (top 30), **(C)** Pro5126-ZmMs7-S11-vs-WT-S11 (top 30).

**Differential genes are mainly concentrated in the biosynthetic pathways related to the formation of the tapetum:** The differentially expressed gene may be involved in the biosynthesis and metabolic processes in biological pathways, as well as in the synthesis of cell membranes, membranes, and cell walls, and the molecular function of lipid binding (Pro5126-ZmMs7-S6-vs-WT-S6, during the formation period of trichomes) (**Figure 10A**). The differential gene may be involved in the biosynthesis and metabolic processes in biological pathways, as well as in the synthesis of cell vacuoles and plastids, and the molecular function of lipid binding (Pro5126-ZmMs7-S11-vs-Pro5126-ZmMs7-S6, mature stage vs. trichome formation stage) (**Figure 10B**); The differential gene may also participate in the biosynthesis and metabolic processes in biological pathways, as well as in the synthesis of cell cytoskeleton and plasma membrane, and the molecular function of lipid binding (Pro5126-ZmMs7-S11-vs-WT-S11, mature stage) (**Figure 10C**). In conclusion, it is evident that the differentially expressed genes are primarily concentrated in the biosynthetic pathways associated with wool layer formation, as well as in the pathways involved in cell membrane, cell wall, and lipid synthesis.

**Figure 11:**
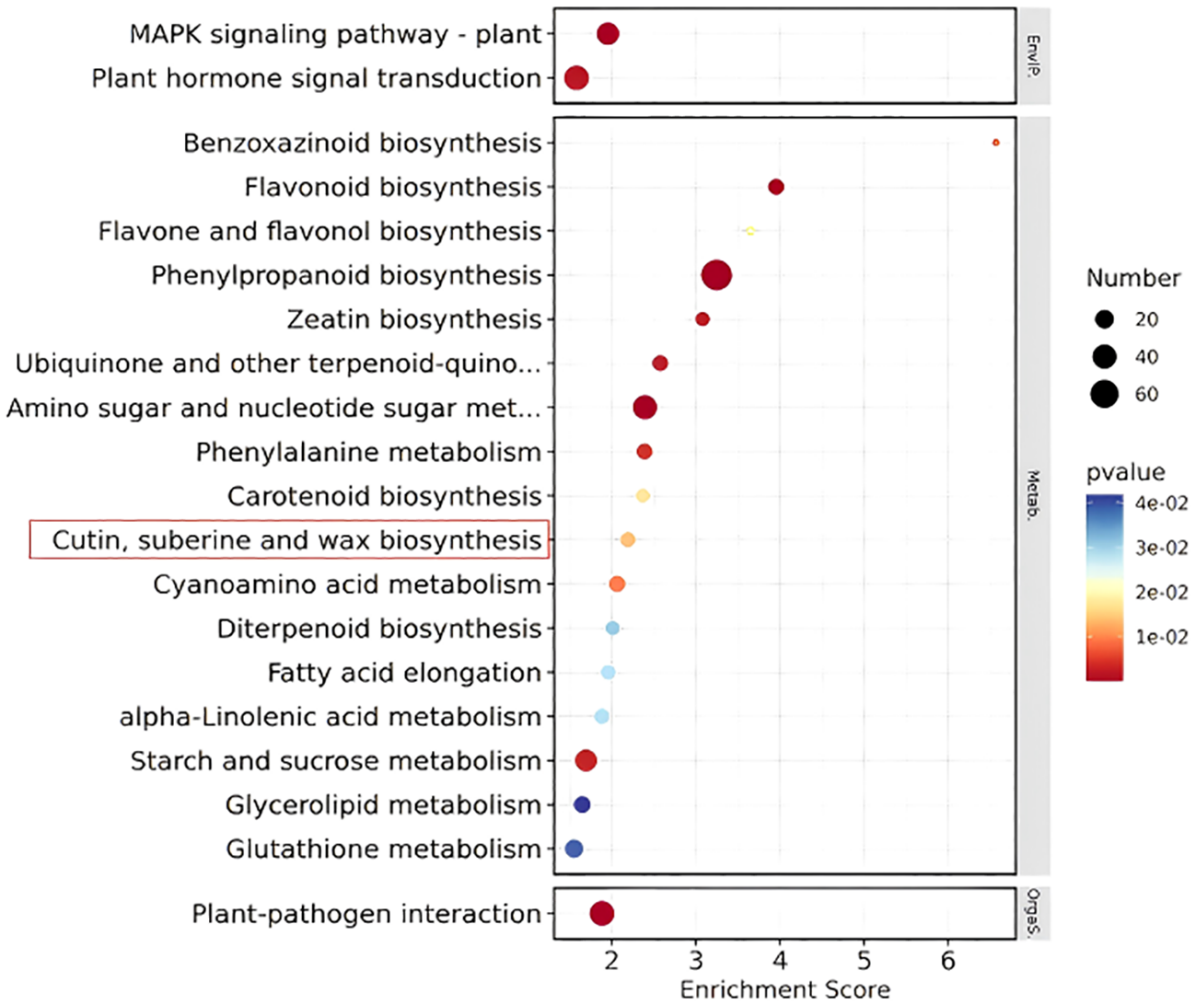
Differential gene KEGG enrichment bubble plot of Pro5126-ZmMs7-S6-vs-WT-S6 (top 20).

**KEGG enrichment bubble chart:** The KEGG enrichment bubble chart (**Figure 11**) of differentially expressed genes from Pro5126-ZmMs7-S6-vs-WT-S6 (top 20) reveals a close association between the normal development pathways of rice fertility and the epidermis (including cuticle, sporopollenin, and wax biosynthesis) (highlighted in red box).

**Figure 12:**
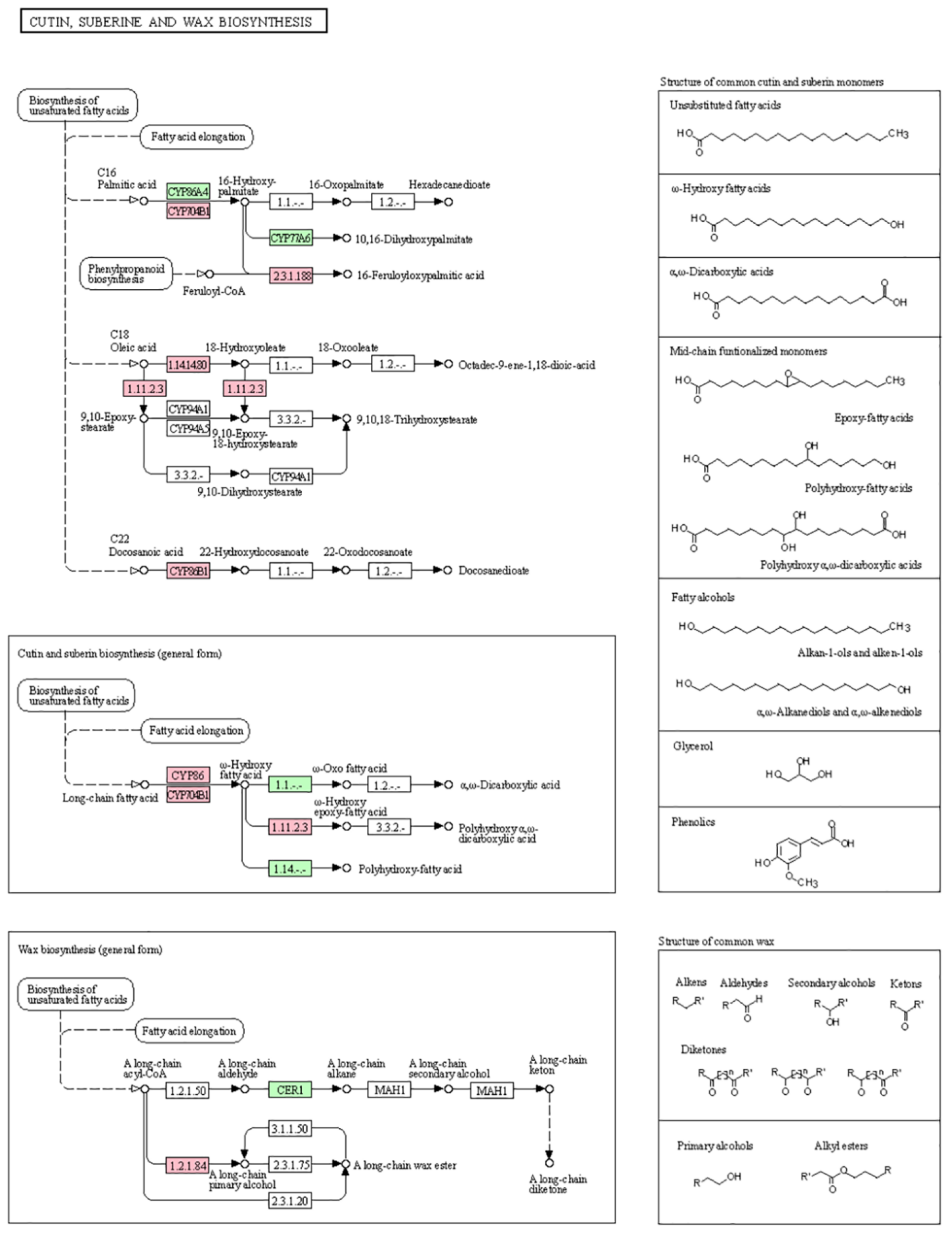
KEGG pathway diagram of Pro5126-ZmMs7-S6-vs-WT-S6 Dots represent nodes in the pathway, which are mainly composed of gene expression products (proteins). Pink represents up-regulated genes, and light blue represents down-regulated genes. Dark green indicates that the corresponding gene is both up-regulated and down-regulated. When there is no color in the pathway, it is equivalent to the union of this pathway in all species.

**KEGG pathway diagram of Pro5126-ZmMs7-S6-vs-WT-S6:** The synthesis and transport of sporopollenin, pollen exine and cuticular wax precursors affect the normal programmed cell death of the tapetum layer: The KEGG pathway diagram of Pro5126-ZmMs7-S6-vs-WT-S6 in **Figure 12** reveals that the differentially expressed genes regulate the formation of unsaturated fatty acids, affecting the synthesis of C16 palmitic acid. This results in the upregulation of *LOG_Os10g34480* (FC=21.75) and *LOG_Os01g63540* (FC=109.9) from the CYP86 subfamily (related to cuticle synthesis), as well as the long-chain fatty acid omega-hydroxylase gene CYP704B1 (*LOG_Os03g07250*, FC=5.232, related to sporopollenin synthesis). Ultimately, this affects the formation of cuticle and sporopollenin. The differentially expressed gene simultaneously regulates the upregulation of wax synthesis genes *LOG_Os04g28620* (FC=6.672) and *LOG_Os03g07140* (FC=16.35), affecting the synthesis of long-chain waxes, ultimately impacting the normal development of the cuticle.

**The validation results are consistent with the differential gene expression in the transcriptome:** Based on the reference genes (*ACT* and *UBQ*), the relative expression levels of *LOC_Os06g06750* are 0.5366 and 0.6257, indicating that its expression is low and lower than the reference genes (Pro5126-ZmMs7-S6-vs-WT-S6). The relative expression levels of *LOC_Os01g66030* and *LOC_Os03g11614* are 3.2179 and 3.5064, 2.2453 and 2.4392, respectively, indicating their relatively high expression levels, far exceeding the reference value (Pro5126-ZmMs7-S11-vs-Pro5126-ZmMs7-S6). Based on the endogenous reference gene ACT, the relative expression levels of *LOC_Os12g03822*, *LOC_Os01g66030*, and *LOC_Os03g11614* are 0.3173, 0.5741, and 0.5004 (Pro5126-ZmMs7-S11-vs-WT-S11). Based on the reference gene *UBQ*, the relative expression level of *LOC_Os12g03822* is 0.4369 (Pro5126-ZmMs7-S11-vs-WT-S11) (**Supplementary Figure 2**). This validation result is consistent with the above differential gene expression in the transcriptome.

#### 3.2.4 Interactions of ZmMs7 with Fertility-related Genes in The Tapetum Layer

**Construction of BD vector:** Based on the CDS sequence of the bait gene ZmMs7, a BD vector named pGBKT7-PHD was constructed using gene synthesis method. The sequencing results are as follows: 5’-GAATTCGACTGCGCGTGCGGAGCGGATGACGACGACGGGGAGCGCATGGCGTGCTGCGACATCTGCGAGGCGTGGCAGCACACCCGGTGCGCGGGGATCAAGGACACCGACGACGCCCCGCACGTCTTCGTCTGCAACCGCTGCGACTAAGGATCC-3’.

**The pGBKT7-PHD shows no self-activation phenomenon on the QDO/X/A medium:** The pGBKT7-53 Control Vector and pGADT7-T Control Vector showed clone growth and turned blue on QDO/X/A medium, indicating the success of the positive control experiment (**Supplementary Figure 3**). The pGBKT7-Lam Control Vector and pGADT7-T Control Vector showed no growth on QDO/X/A medium, indicating successful negative control experiments (**Supplementary Figure 4**). The BD gene pGBKT7-PHD+pGADT7 shows blue growth on DDO/X medium, indicating successful transformation of the pGBKT7-PHD plasmid into yeast strain. However, it does not grow on QDO/X/A medium. This suggests that pGBKT7-PHD is suitable for subsequent screening experiments (**Supplementary Figure 5**).

**A total of 87 candidate positive clones were identified from the screening library:** The pGBKT7-PHD was mated with the yeast cDNA library of rice (AD-XYS) for library screening. As a result, there were 93 blue clones on the QDO/X/A selection plates (transformation efficiency: total number of transformants >2x10^6cfu) (**Supplementary Figure 6A, B**). Blue clones were picked from QDO/X/A selection plates and transferred to QDO/X/A screening plates for secondary screening. A total of 87 candidate positive clones grew blue, which were subsequently subjected to next-generation sequencing (**Supplementary Figure 6C**).

**284 genes were detected in the positive clone:** Positive clones were identified through second-generation sequencing, detecting a total of 284 genes. Combined with transcriptome experiments, 4 positive clones related to rice fertility were selected from the 284 genes for validation experiments (**Table 2**).

**Table 2:** Information on four fertility-related genes.

Gene	Genetic information	E_value	Genebank
pGADT7-LOC_Os03g11614_03	MADS-box transcription factor 1 [Oryza sativa Japonica Group]	2E-166	XP_015628585.1
pGADT7-LOC_Os06g06750_04	MADS-box transcription factor 5 [Oryza sativa Japonica Group]	8E-164	XP_015641896.1
pGADT7-LOC_Os12g03822_01	Glutamate-rich WD repeat-containing protein 1, putative, expressed [Oryza sativa Japonica Group]	8E-164	XP_015641896.1
pGADT7-LOC_Os12g03822_02	glutamate-rich WD repeat-containing protein 1 [Oryza sativa Japonica Group]	0	XP_015619858.1

**Four positive clones related to reproductive development interact with pGBKT7-PHD:** Four candidate positive clones were selected for activation and recovery, including yeast shaking culture, plasmid extraction, transformation into Escherichia coli DH5α, extraction of AD plasmids, and one-to-one validation with pGBKT7-PHD. Four positive clones were obtained, revealing that pGADT7-LOC_Os12g03822_01 (*LOC_Os12g03822_02* and *LOC_Os12g03822_01* are the same gene), pGADT7-LOC_Os03g11614_03, and pGADT7-LOC_Os06g06750_04 all exhibited positive growth on DDO/X and QDO/X/A media. This indicates interaction with pGBKT7-PHD (**Supplementary Figure 7**).

**Figure 13:**
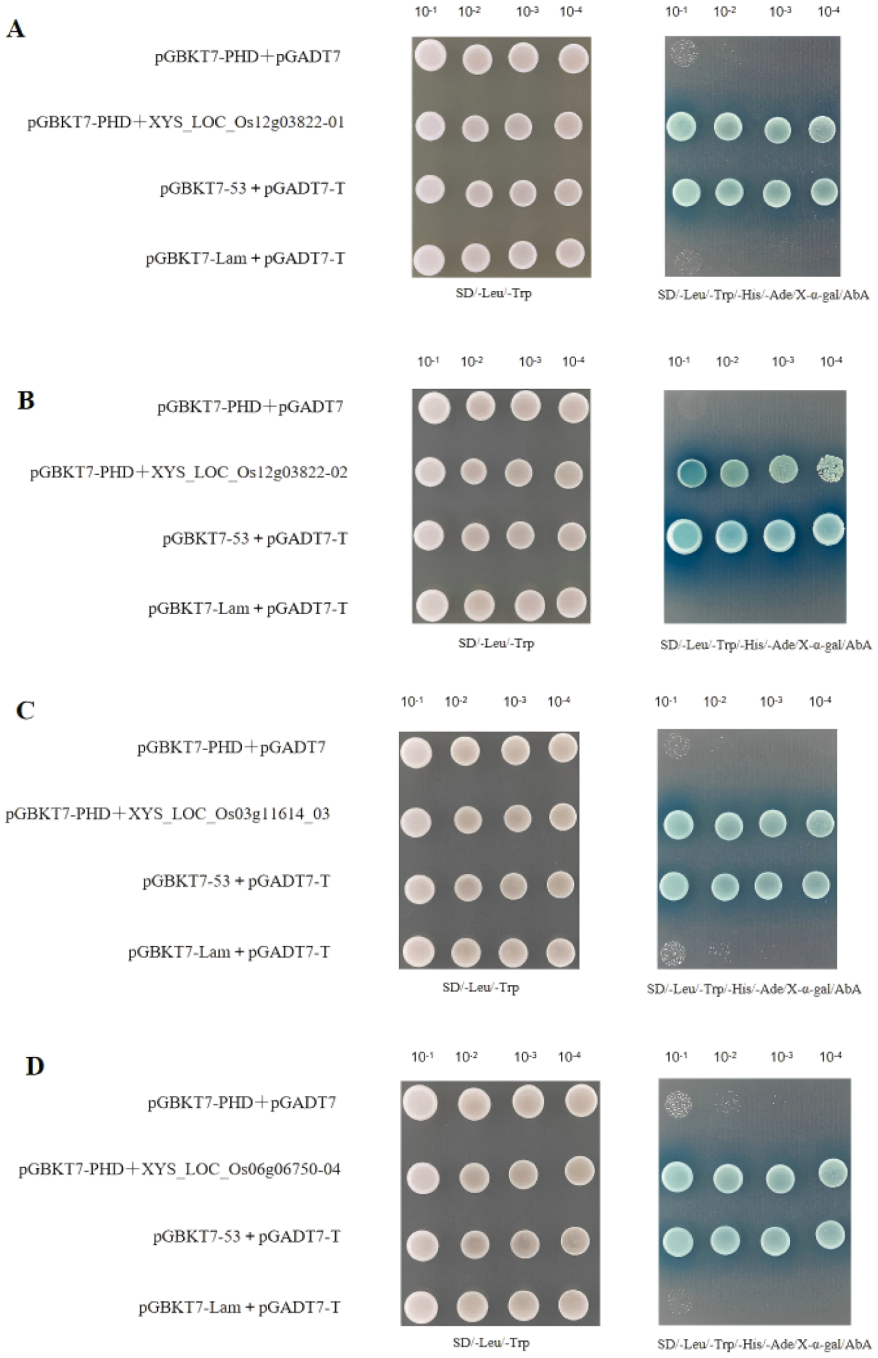
Validation of candidate positive clones **(A)** pGBKT17-PHD+XYS_LOC_Os12g03822_01 for validation on agar plates, **(B)** pGBKT17-PHD+XYS_LOC_Os12g03822_02 for validation on agar plates, **(C)** pGBKT17-PHD+XYS_LOC_Os03g11614_03 for validation on agar plates, **(D)** pGBKT17-PHD+XYS_LOC_Os06g06750_04 for validation on agar plates.

**One-on-one verification:** Both pGBKT17-PHD-53 and pGADT7-T showed yeast colonies, indicating successful positive controls. However, there was no interaction in SD/Leu/Trp/-His/-Ade/X-α-gal/AbA, indicating successful negative controls. No interaction was observed in SD/Leu/Trp/-His/-Ade/X-α-gal/AbA, indicating no self-activation. Yeast colonies of pGBKT17-PHD+XYS_LOC_Os12g03822_01, pGBKT17-PHD+XYS_LOC_Os12g03822_02, pGBKT17-PHD+XYS_LOC_Os03g11614_03, and pGBKT17-PHD+XYS_LOC_Os06g06750_04 grew on different antibiotic media, consistent with the results of one-on-one validation (**Figure 13**). Further evidence has demonstrated that the proteins encoded by pGADT7-LOC_Os03g11614, pGADT7-LOC_Os06g06750, and pGADT7-LOC4332059 can all interact with pGBKT7-PHD.

## 4 Discussion

### 4.1 A Simple and versatile biotechnology for multiple crops

By transforming recessive male sterile plants related to pollen lethal genes and marker genes, transgenic maintainer lines are obtained. During self-pollination, this maintainer line will reproduce maintainer lines and male sterile lines (Chang et al., 2016). Previous researchers put forward two strategies: one is to transform the restorer genes related to seed color selection genes into male sterile plants; the other is to introduce the fertility restorer genes related to pollen lethal genes into male sterile plants (Stetter et al., 2016; Zou et al., 2017). The cross between restorer and male-sterile lines results in 50% male-sterile seeds and 50% fluorescent seeds through heterozygous pollination, which can be distinguished by seed color. In this experiment, the Pro5126-ZmMs7-DsRed system produced two types of F1 hybrid seeds: red fluorescent transgenic dominant male sterile seeds and normal colored non-transgenic fertile seeds. For crops like corn, sunflower, and rapeseed that undergo cross-pollination, both F1 hybrid seed types can be utilized flexibly in agricultural production across different countries. In nations where genetically modified crops are banned, non-transgenic male-sterile hybrid seeds can be selected for planting. Conversely, in countries permitting genetically modified crops, both F1 hybrid seed types may be mixed and sown together (Wang et al., 2020; Chalvin et al., 2021). Although 50% of the transgenic dominant male-sterile F1 plants are male-sterile, the other 50% of non-transgenic male-fertile F1 siblings can provide sufficient pollen for pollinating the male-sterile plants, ensuring no impact on field yield. For self-pollinating crops like rice, sorghum, and millet, nearly 50% of non-transgenic male-fertile hybrid seeds can be selected and planted. Compared to CMS and other biotechnology-based male sterility systems, this system offers several advantages: This research is based on the heterologous precocious expression of *ZmMs7*, demonstrating high stability across different genetic backgrounds; unlike seed production technology and multi-control systems, it is not constrained by the absence of GMS mutants or fertility restoration genes in many crops. In conclusion, the Pro5126-ZmMs7-DsRed system is a simple, cost-effective, and efficient biotechnology suitable for various crops.

### 4.2 Establishment of a novel dominant nuclear male-sterile line in rice through heterologous expression of ZmMs7

Based on the function and regulation timing of infertility genes, conventional nuclear infertility genes can be classified into three categories: those active during the growth stage of microspore mother cells, those in the tapetum growth stage, and those involved in pollen sac and exine formation (Shi et al., 2020). The tapetum is a specialized tissue within the anther’s four-layered wall structure that directly contacts microspores, providing essential enzymes and sporopollenin for their development (Jung et al., 2006). In rice, the nucellar epidermis supplies nutrition to microspore mother cells and plays a key role in synthesizing callose enzymes while decomposing tetrad callose outer walls. It also synthesizes recognition proteins and sporopollenin for the pollen wall, enhancing pollen stability (Ji et al., 2015). During anther development post-meiosis, the tapetum undergoes programmed cell death (PCD), essential for forming the anther epidermis and pollen exine. Proper degradation of the tapetum is crucial; premature or delayed PCD can result in male sterility. This study identified the p5126-ZmMs7-DsRed-YS male sterile mutant, which expresses the *ZmMs7* gene early via promoter p5126 in rice anthers. This alteration disrupts normal PCD in tapetal cells, preventing them from supplying nutrients and structural materials for microspore development, leading to defective pollen exine and complete male sterility. The *PTC1* gene is highly conserved in plants and regulates male development. Its homologs include AtMs1 in Arabidopsis and *ZmMs7* in maize; mutations in these genes lead to stable male sterility. In 2017, the team led by Xiangyuan Wan utilized *ZmMs7* to establish a multi-control male sterility system for maize, effectively addressing reproductive challenges associated with conventional nuclear male sterile lines. This study successfully developed a dominant nuclear male-sterile line of rice (p5126-ZmMs7-DsRed-YS) through the heterologous expression of ZmMs7 from maize, thereby demonstrating its potential for constructing dominant nuclear male-sterile lines across various crop species.

### 4.3 Analysis of the male sterility mechanism of new type dominant nuclear male sterile rice

In a case study on the research of crop hybrid vigor, the utilization of male sterility materials has demonstrated significant commercial value. Jiang successfully cloned three dominant nuclear male sterile genes from identified mutants and elucidated the regulatory transcription factors for male sterility in maize based on CRISPR/Cas9, as well as their functional conservation in plants (Jiang et al., 2021). However, in contrast to the progress in research on cytoplasmic male sterility, which is sensitive to nuclear environment, most studies on dominant nuclear male sterility in rice have only focused on preliminary chromosome localization and cytological analysis. There are few cases of successful cloning of male sterile genes, and the regulatory mechanism for male sterility is also lacking clear explanation (Dong et al., 2021; Lan et al., 2021).

This study analyzed the molecular regulation of *ZmMs7* heterologous expression in rice on its male fertility. As a transcriptional activator, premature expression of *ZmMs7* driven by p5126 altered the expression pattern of a series of genes involved in anther and pollen development in rice, leading to dominant male sterility. Research has revealed that the premature expression of *ZmMs7* driven by p5126 may induce changes in the gene expression pattern, potentially involved in the development of tapetum and pollen exine formation, leading to complete male sterility. The homology of *ZmMs7* has been identified in several plant species, including *AtMs1*, *OsMs1/OsPTC1*, and *HvMs1*. However, the precise molecular mechanisms and direct target genes of these transcription factors remain largely unknown. Many scholars have explored the expression sites and periods of genes through temporal and spatial expression analysis. Previous studies have found that *OsNP1* (male sterility gene in rice) shows specific expression in meiotic stages (stage 7 and stage 8) of anthers based on qPCR results, while it is not expressed in roots, stems, leaves, pistils, and anthers at other developmental stages (Chang et al., 2016). In this study, transgenic plants Pro5126:GUS and ProZmMs7:GUS were found to not express in their roots, stems, leaves, inner glumes, and outer glumes. The transgenic material Pro5126:GUS was observed to express during the 6th (formation of the nucellar epidermis) to 8th (late meiosis) stages of seed development, with the gene expressing in the anthers. It is speculated that the p5126 promoter causes *ZmMs7* to express one stage earlier, leading to male sterility in rice. At the 6th stage of development, differential genes in p5126-ZmMs7 rice are significantly enriched in the normal developmental pathway of the cuticle layer (containing wax, sporopollenin and lipid biosynthesis) closely related to fertility. This leads to up-regulation of *LOG_Os04g28620*, *LOG_Os03g07140*, *LOG_Os10g34480*, *LOG_Os01g63540* and *LOG_Os03g07250*. However, *LOG_Os04g28620* and *LOG_Os03g07140* are important genes for wax synthesis, while *LOG_Os10g34480* and *LOG_Os01g63540* are involved in cuticle synthesis in rice cuticle layer. The long-chain fatty acid omega-hydroxylase gene CYP704B1 (*LOG_Os03g07250*) is also a key gene for sporopollenin synthesis in plant membranes. This indirectly or directly leads to the up-regulation of rice pollen fertility genes *LOC_ Os12g03822* and down-regulation of *LOC_Os06g06750* and *LOC_Os03g11614*. Y2Y experiments were conducted on these three genes, with results showing that pGADT7-LOC_Os12g03822, pGADT7-LOC_Os03g11614 and pGADT7-LOC_Os06g06750 interact with pGBKT7-PHD. It is speculated that the introduction of p5126-ZmMs7 leads to the premature expression of *ZmMs7* in rice, resulting in abnormal expression of genes related to the synthesis of cuticle, pollen wall and wax. This in turn affects the normal programmed cell death of tapetum, leading to abnormal expression of male fertility-related genes in anthers, ultimately causing sterility in the material p5126-ZmMs7-DsRed-YS.

### 4.4 The new dominant nuclear sterile line has promising prospects for application

Due to the fact that the sterility of the second generation hybrid rice is controlled by mendelian factors, its reproductive ability is only affected by changes in light, temperature, and other environmental factors. There is no need to worry about the genetic type of the restorer cells, thus breaking free from the constraints of “restoration-keeping” relationship (Okayama et al., 1991). Nevertheless, the conversion of restorer lines and other fertile rice varieties into the sterile lines for second-generation hybrid rice is not an easy task, as the threshold for their fertility transformation varies due to different genetic backgrounds of rice. In previous studies, researchers used gene editing technology to induce mutations in the *TMS5* gene of eight different genetic backgrounds of rice, including the Restorer line Wuxiang S and five CMS lines. However, only two male-sterile lines showed a lowered fertility conversion threshold below 24°C (Tan et al., 2020; Fang et al., 2022). Therefore, even though the male-sterile lines of the second-generation hybrid rice have been freed from the constraints of the “restoration and maintenance” genetic background, they still face limitations imposed by the genetic background of the threshold for fertility conversion. This means that developing a second-generation hybrid rice male-sterile line with an ideal critical condition for fertility transformation remains an extremely challenging task.

The research on the p5126-ZmMs7-DsRed-YS male sterile line shows that it is not restricted by cytoplasmic genotype and does not require consideration of the genetic background for fertility conversion. It is well known that male sterility is the most effective method to ensure cross-pollination and produce pure hybrid seeds. According to reports, the *ZmMs7* gene and its mutant variant ms7-6007 have been utilized in our laboratory to develop a multi-control male sterility system, which can be used for maintaining and breeding maize cytoplasmic male sterile lines. In this study, we tested the genetic stability of the p5126-ZmMs7-DsRed-YS male sterile line and found that the segregation in the F1 population was close to the expected 1:1 ratio. Furthermore, their corresponding phenotypes were completely matched.

### 4.5 The limitations of the experiment

Although dominant nuclear male sterile lines play a significant role in plant breeding, their application also requires comprehensive consideration of potential limitations and challenges. For instance, extensive reliance on a single sterile line may increase the population’s susceptibility to diseases and environmental changes; the process of producing sterile lines and maintainer lines can be complex and time-consuming; and the involvement of transgenic technology may raise public concerns about food safety and environmental impacts.

Although this study provides significant insights into candidate genes and their interacting proteins, the absence of some key experimental validation methods (such as BiFC, Co-IP, Pull-down assay, and transgenic analysis) may introduce certain uncertainties regarding the reliability and applicability of the results. Future research should incorporate these experiments to enhance the depth and breadth of the study’s conclusions.

## 5 Conclusions

In this paper, we investigated the use of the male fertility control gene *ZmMs7* previously reported in maize, with “NG 9108” as the background material. We successfully constructed a visual selection dominant sterile carrier system Pro5126-ZmMs7-DsRed, providing a new approach for creating novel sterile lines in rice. The anther of this dominant nuclear male-sterile line is relatively small and lacks pollen, but the female fertility is normal, and the plant growth is not affected. The F1 show a stable 1:1 ratio of fluorescent to non-fluorescent seeds, and the trait can be stably inherited. The study has demonstrated that the heterologous gene *ZmMs7*, in combination with the promoter p5126, causes abnormal expression of the rice pollen fertility genes *LOC_Os12g03822* (*RIP1*), *LOC_Os06g06750* (*OsMADS5*), and *LOC_Os03g11614* (*OsMADS1* and *LHS1*). Furthermore, it has been observed that the proteins expressed by these three fertility genes interact with the protein encoded by *ZmMs7*, leading to premature degradation of the rice tapetum and ultimately resulting in dominant male sterility in rice. This male-sterile line resolves the traditional method of manual emasculation and hybridization in breeding, saving time and being both simple and efficient. In addition, the selection of dominant sterile line intermediates for recurrent hybridization can be based on whether the seeds are fluorescently labeled. Non-fluorescent signals indicate fertile seeds, which can be used to breed target strains with excellent traits in separate generations and serve as important tools for utilizing hybrid vigor. Simultaneously elucidating the molecular regulatory mechanism of male reproductive development in rice provides a theoretical basis and technical support for the creation and utilization of new sterile germplasm resources. This also serves as a reference for the production and application of dominant nuclear sterile materials in other plants.

## Data Availability

The datasets presented in this study can be found in online repositories. The names of the repository/repositories and accession number(s) can be found below: https://www.jianguoyun.com/p/DZPiDG4Qg8umDBjgyOoFIAA, 1 https://www.jianguoyun.com/p/DcT4CEkQg8umDBjiyOoFIAA, 1 https://www.jianguoyun.com/p/DeDyLtQQg8umDBjkyOoFIAA, 1.
