# Supplementary material for: The molecular mechanism and utilization of ZmMs7-mediated dominant nuclear sterility in Oryza sativa L
Source: Front Plant Sci. 2025 Apr 29;16:1572721. doi: 10.3389/fpls.2025.1572721 (PMC12069387; doi:10.3389/fpls.2025.1572721)
Supplement: Supplementary file 1 [file DataSheet1.pdf]

Supplementary material

Table 1. Preparation of 100 mm flat plate.

	SD/-Trp	SD/-Trp/X-α-Gal	SD/-Trp/X-α-Gal/AbA	SD/-Trp/-Leu/X-α-Gal
1	-	-	-	50 μl
2	-	-	-	50 μl
3	50 μl	50 μl	50 μl	-
4	50 μl	-	-	-

Table 2. Expected results (BD does not have self-activation).

Raction	Type of culture medium	Are there positive clones	Clone color
1（Positive control）	SD/-Trp/-Leu/X-α-Gal	Yes	Blue
2（Negative control）	SD/-Trp/-Leu/X-α-Gal	Yes	White
3（BD self-activation detection）	SD/-Trp	Yes	White
4（BD self-activation detection）	SD/-Trp/X-α-Gal	Yes	White
5（BD self-activation detection）	SD/-Trp/X-α-Gal/ABA	No	Blank
6（Blank BD Carrier）	SD/-Trp	Yes	White

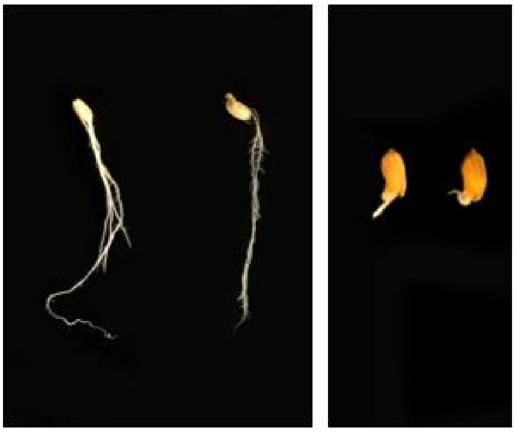

Figure 1. *GUS* staining analysis of roots and germinated seeds of transgenic rice Pro5126:GUS and ProZmMs7:GUS after 10 days of growth.

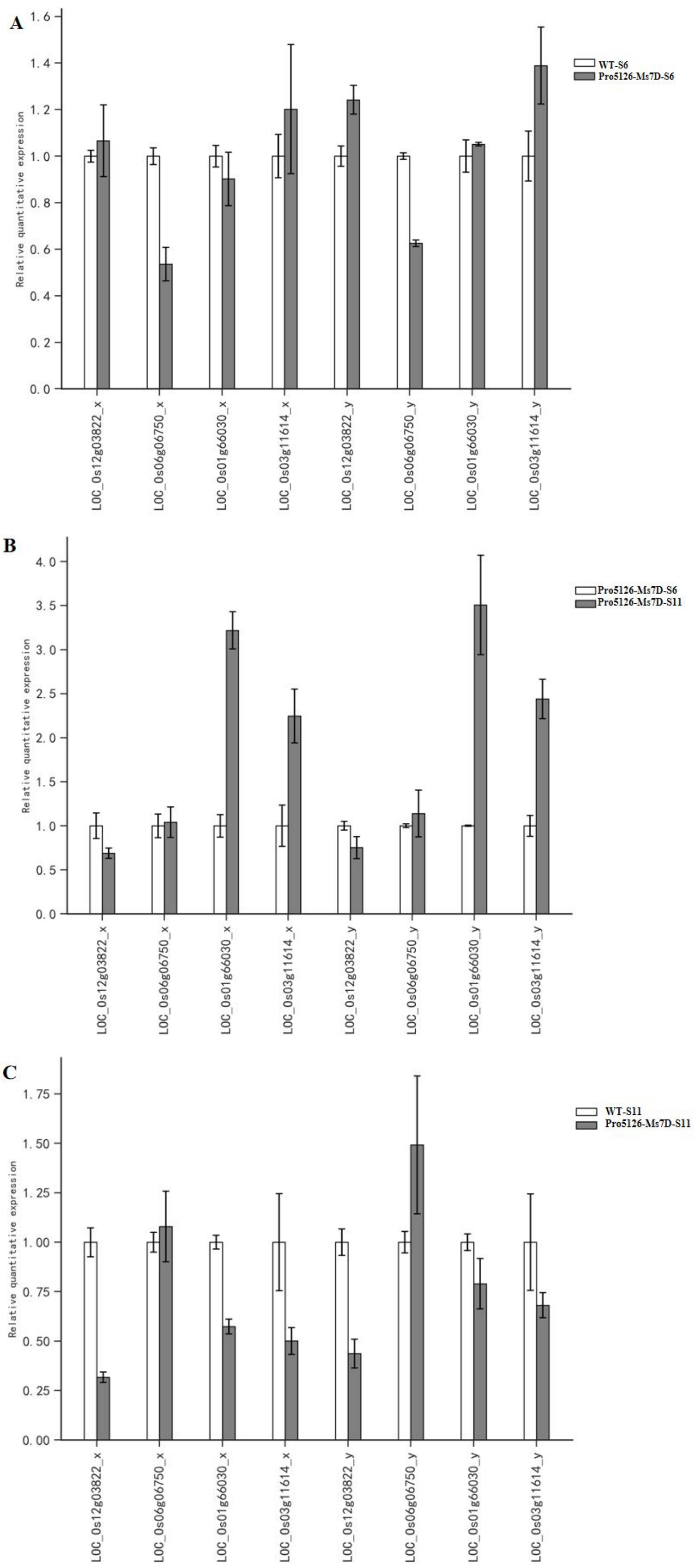

Figure 2. Differential gene expression histogram A: Pro5126-ZmMs7-S6-vs-WT-S6, B: Pro5126-ZmMs7-S11-vs-Pro5126-ZmMs7-S6, C: ro5126-ZmMs7-S11- vs-WT-S11, _x represents the internal parameter ACT, _y represents the internal parameter UBQ.

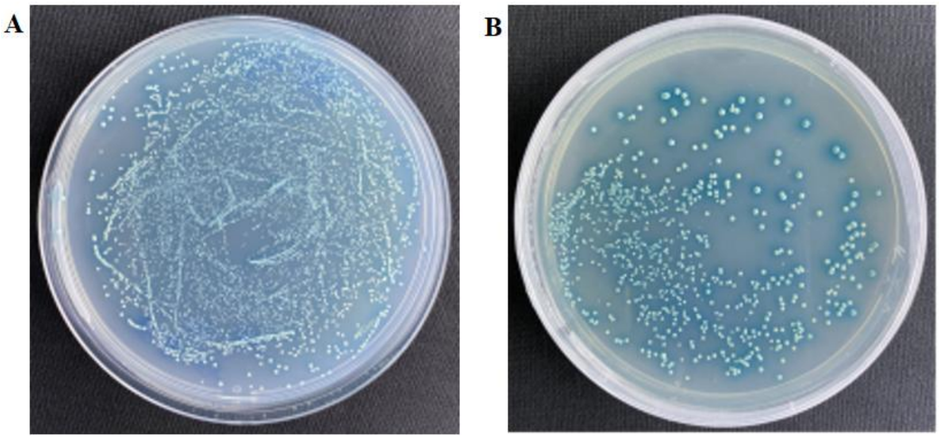

Figure 3. Positive control for the experiment of pGBKT7-PHD self-activation A: DDOX cultivation medium, B: QDOXA cultivation medium.

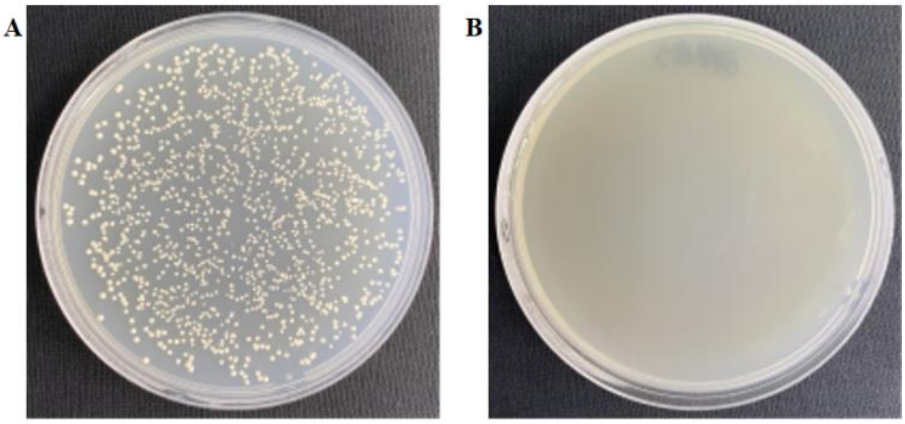

Figure 4. Negative control for the experiment of pGBKT7-PHD self-activation A: DDO/X cultivation medium, B: QDO/X/A cultivation medium.

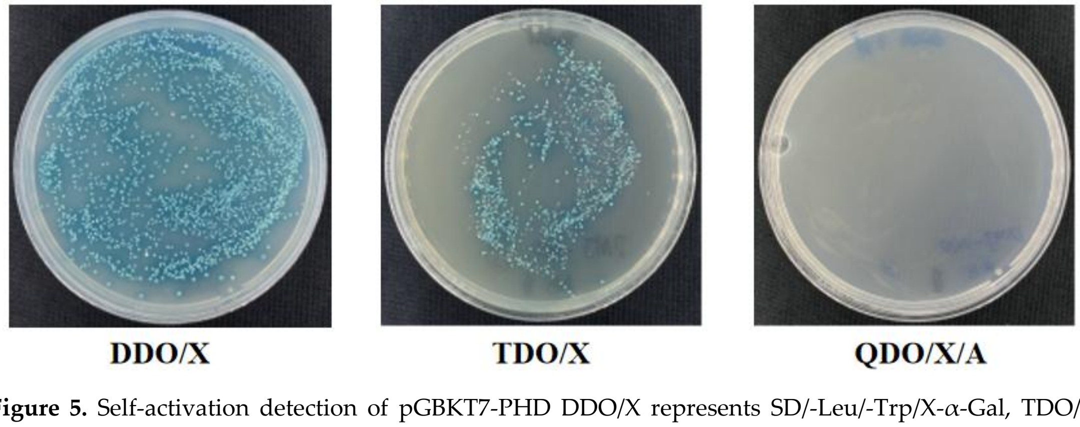

Figure 5. Self-activation detection of pGBKT7-PHD DDO/X represents SD/-Leu/-Trp/X-α-Gal, TDO/X represents SD/-Leu/-Trp/-His/X-α-Gal, QDO/ X/A stands for SD/-Leu/-Trp/-His/-Ade/X-α-Gal/AbA.

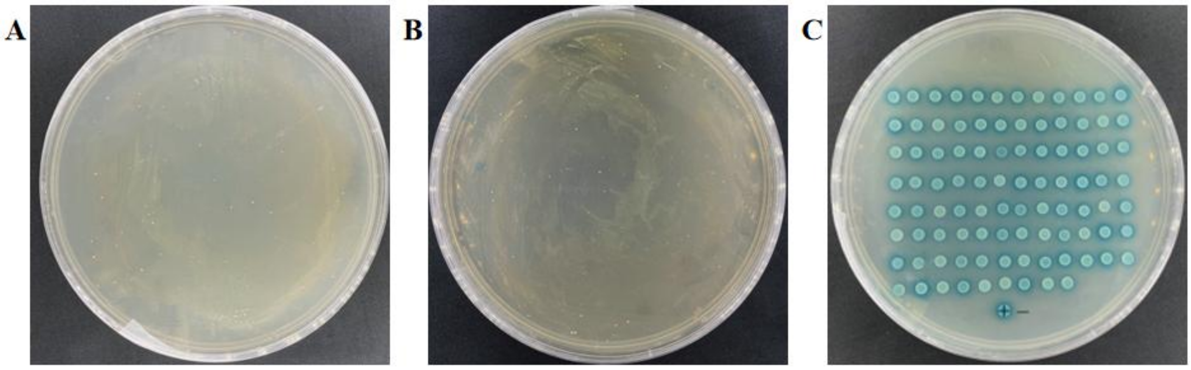

Figure 6. Positive clones on the screening library plate A and B: Some positive clones on the primary screening plate (QDO/X/A), C: Positive clones on the rescreening plate (QDO/X/A).

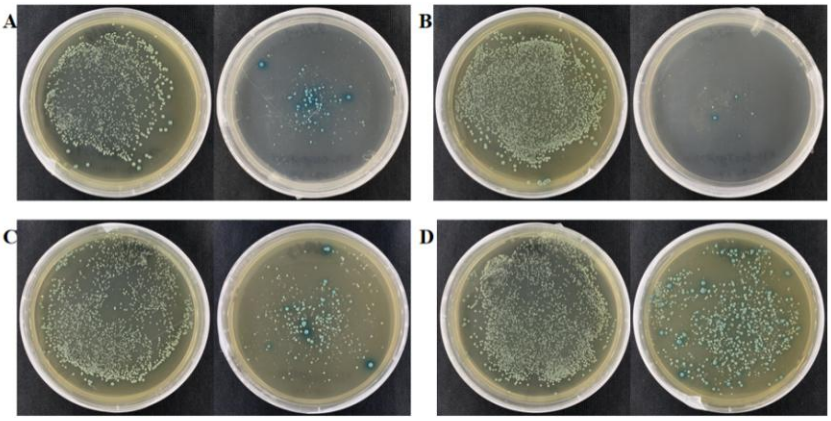

Figure 7. One-to-one verification results of four positive clones A: pGADT7-LOC_Os03g11614_03 positive clone (Left image: DDO/X medium. Right image: QDO/X/A medium), B: pGADT7-LOC_Os06g06750_04 positive clone (Left image: DDO/X medium. Right image: QDO/X/A medium), C: pGADT7-LOC_Os12g03822_02 positive clone (Left image: DDO/X medium. Right image: QDO/X/A medium), D: pGADT7-LOC_Os12g03822_01 positive clone (Left image: DDO/X medium. Right image: QDO/X/A medium).
